# Crystal structure of (3*E*)-3-(2,4-di­nitro­phen­oxy­meth­yl)-4-phenyl­but-3-en-2-one

**DOI:** 10.1107/S1600536814018819

**Published:** 2014-08-23

**Authors:** Ignez Caracelli, Stella H. Maganhi, Paulo J. S. Moran, Bruno R. S. de Paula, Felix N. Delling, Edward R. T. Tiekink

**Affiliations:** a BioMat-Departmento de Física, Universidade Federal de São Carlos, 13565-905 São Carlos, SP, Brazil; b Instituto de Química, Universidade Estadual de Campinas, CP 6154, 13083-970 Campinas, SP, Brazil; c Departmento de Qímica, Universidade Federal de São Carlos, 13565-905 São Carlos, SP, Brazil; d Department of Chemistry, University of Malaya, 50603 Kuala Lumpur, Malaysia

**Keywords:** crystal structure, hydrogen bonding, N⋯O inter­actions

## Abstract

In the title compound, C_17_H_14_N_2_O_6_, the conformation about the C=C double bond [1.345 (2) Å] is *E*, with the ketone moiety almost coplanar [C—C—C—C torsion angle = 9.5 (2)°] along with the phenyl ring [C—C—C—C = 5.9 (2)°]. The aromatic rings are almost perpendicular to each other [dihedral angle = 86.66 (7)°]. The 4-nitro moiety is approximately coplanar with the benzene ring to which it is attached [O—N—C—C = 4.2 (2)°], whereas the one in the *ortho* position is twisted [O—N—C—C = 138.28 (13)°]. The mol­ecules associate *via* C—H⋯O inter­actions, involving both O atoms from the 2-nitro group, to form a helical supra­molecular chain along [010]. Nitro–nitro N⋯O inter­actions [2.8461 (19) Å] connect the chains into layers that stack along [001].

## Related literature   

For background to biotransformations mediated by *Saccharomyces cerevisiae*, see: Rodrigues *et al.* (2004 ▶); de Paula *et al.* (2013 ▶). For a related structure, see: Zukerman-Schpector *et al.* (2014 ▶). For inter­actions between nitro groups, see: Daszkiewicz (2013 ▶).
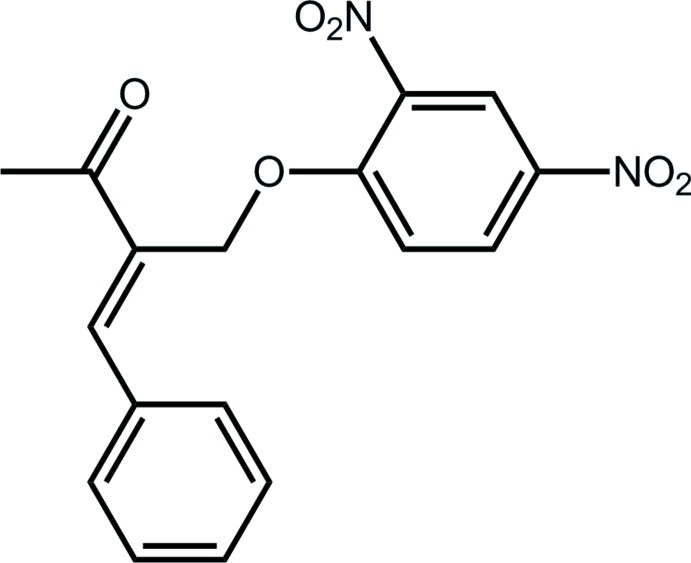


## Experimental   

### Crystal data  

C_17_H_14_N_2_O_6_
*M* *_r_* = 342.30
Monoclinic, 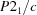

*a* = 12.8459 (6) Å
*b* = 7.6983 (4) Å
*c* = 19.4283 (8) Å
β = 122.254 (2)°
*V* = 1624.82 (14) Å^3^
*Z* = 4
Mo *K*α radiation
μ = 0.11 mm^−1^
*T* = 290 K
0.66 × 0.45 × 0.28 mm

### Data collection  

Bruker Kappa APEXII CCD diffractometer
Absorption correction: multi-scan (*SADABS*; Sheldrick, 1996 ▶) *T*_min_ = 0.699, *T*_max_ = 0.745
10435 measured reflections
2957 independent reflections
2630 reflections with *I* > 2σ(*I*)
*R*_int_ = 0.019

### Refinement  

*R*[*F* ^2^ > 2σ(*F* ^2^)] = 0.037
*wR*(*F* ^2^) = 0.101
*S* = 1.04
2957 reflections
228 parameters
H-atom parameters constrained
Δρ_max_ = 0.20 e Å^−3^
Δρ_min_ = −0.21 e Å^−3^

### 

Data collection: *APEX2* (Bruker, 2009 ▶); cell refinement: *SAINT* (Bruker, 2009 ▶); data reduction: *SAINT*; program(s) used to solve structure: *SIR97* (Altomare *et al.*, 1999 ▶); program(s) used to refine structure: *SHELXL97* (Sheldrick, 2008 ▶); molecular graphics: *ORTEP-3 for Windows* (Farrugia, 2012 ▶), *QMol* (Gans & Shalloway, 2001 ▶) and *DIAMOND* (Brandenburg, 2006 ▶); software used to prepare material for publication: *MarvinSketch* (ChemAxon, 2010 ▶) and *publCIF* (Westrip, 2010 ▶).

## Supplementary Material

Crystal structure: contains datablock(s) I. DOI: 10.1107/S1600536814018819/hg5406sup1.cif

Structure factors: contains datablock(s) I. DOI: 10.1107/S1600536814018819/hg5406Isup2.hkl

Supporting information file. DOI: 10.1107/S1600536814018819/hg5406Isup3.cml

**Fig. 1.**
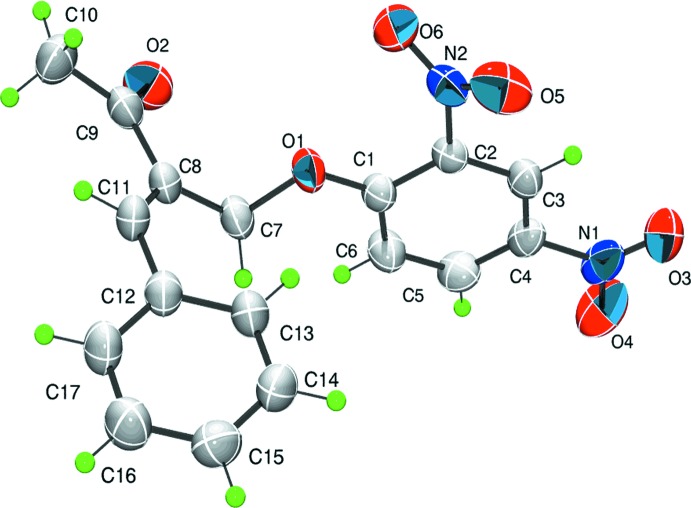
The molecular structure of the title showing the atom-labelling scheme and displacement ellipsoids at the 50% probability level.

. DOI: 10.1107/S1600536814018819/hg5406fig1.tif
The mol­ecular structure of the title showing the atom-labelling scheme and displacement ellipsoids at the 50% probability level.

**Fig. 2.**
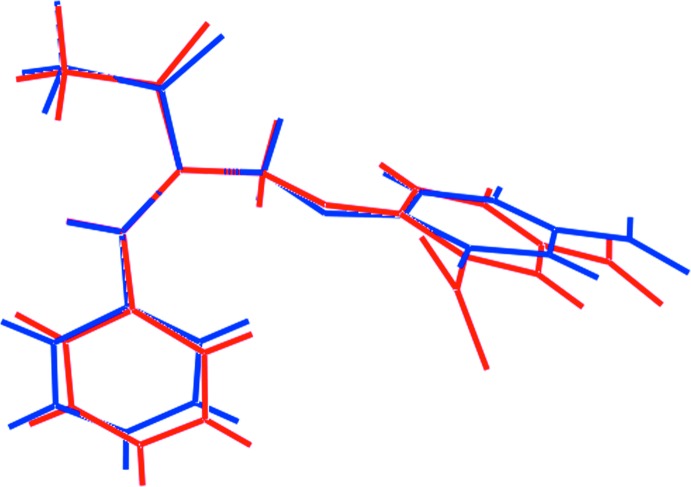
Overlay diagram of (I), red image, with inverted (II), blue image, drawn so that the C═C—C(phenyl) atoms are overlapped.

C C . DOI: 10.1107/S1600536814018819/hg5406fig2.tif
Overlay diagram of (I), red image, with inverted (II), blue image, drawn so that the C=*C*—*C*(phen­yl) atoms are overlapped.

**Fig. 3.**
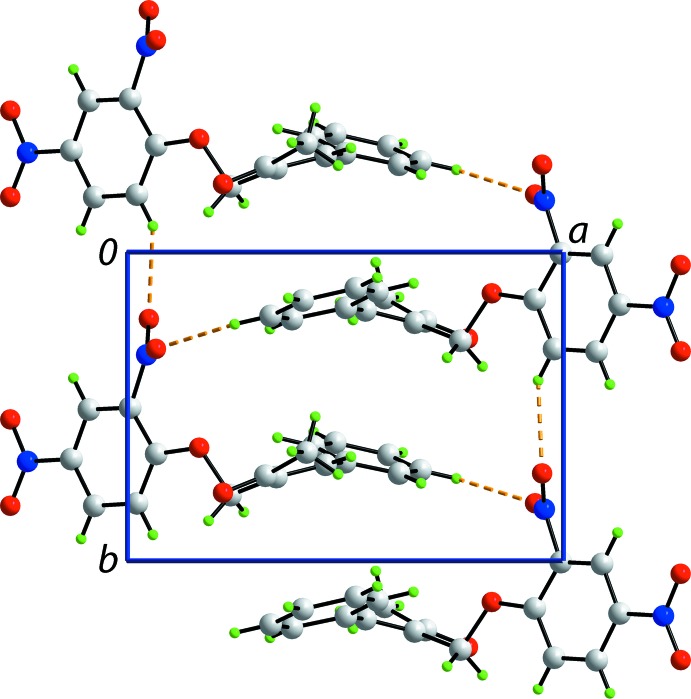
A view of helical supramolecular chain along [0 1 0]. The C—H···O contacts are shown as orange dashed lines.

**Fig. 4.**
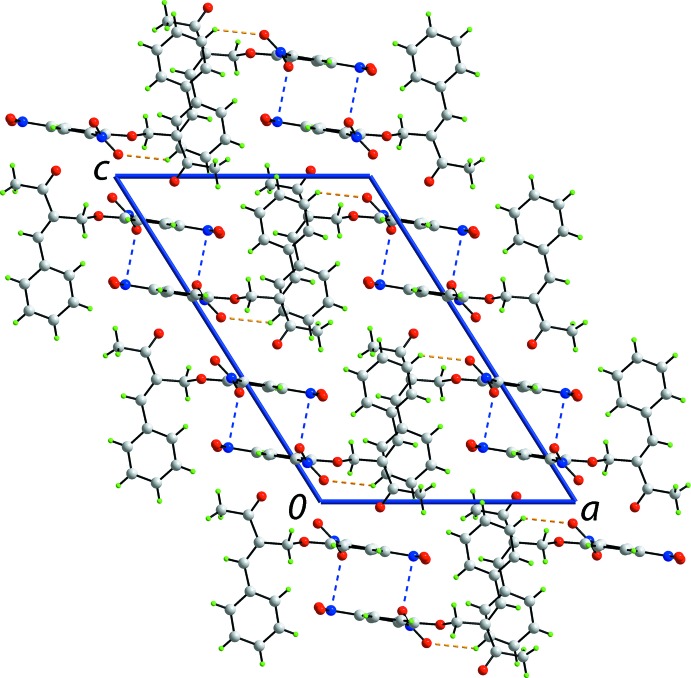
A view of unit-cell contents in projection down the b axis. The C—H···O and N···O contacts are shown as orange and blue dashed lines, respectively.

. DOI: 10.1107/S1600536814018819/hg5406fig3.tif
A view of helical supra­molecular chain along [0 1 0]. The C—H⋯O contacts are shown as orange dashed lines.

b . DOI: 10.1107/S1600536814018819/hg5406fig4.tif
A view of unit-cell contents in projection down the *b* axis. The C—H⋯O and N⋯O contacts are shown as orange and blue dashed lines, respectively.

CCDC reference: 1020026

Additional supporting information:  crystallographic information; 3D view; checkCIF report

## Figures and Tables

**Table 1:** Hydrogen-bond geometry (Å, °)

*D*—H⋯*A*	*D*—H	H⋯*A*	*D*⋯*A*	*D*—H⋯*A*
C6—H6⋯O5^i^	0.93	2.41	3.1375 (18)	135
C16—H16⋯O6^ii^	0.93	2.58	3.292 (3)	134

Symmetry codes: (i) 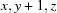; (ii) 
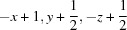
.

## supplementary crystallographic information

### S2. Structural commentary

In the context of the study of biotransformation reactions mediated by *Saccharomyces cerevisiae*, such as the bio-reduction of α-haloketones and enones (Rodrigues *et al.*, 2004), the title compound, (3*E*)-3-(2,4-di­nitro­phen­oxy­methyl)-4-phenyl­but-3-en-2- one, (I), as well as its 4-nitro­phenyl­methyl analogue, i.e. (3*E*)-3-(4-nitro­phen­oxy­methyl)-4-phenyl­but-3-en-2-one, (II), (Zukerman-Schpector *et al.* 2014), were synthesised to be used as substrates in order to compare their behaviour with that of the 3-halo­methyl-4-phenyl-3-buten-2-ones analogues (de Paula *et al.*, 2013). Herein, the crystal structure determination and spectroscopic details of (I) are described.

In (I), the conformation about the C═C double bond [1.345 (2) Å] is *E*. The ketone moiety almost co-planar [C11–C8–C9–C10 torsion angle = 9.5 (2) °] with the double bond but the phenyl ring is twisted [C8–C11–C12–C17 = -150.23 (16)°]. The aromatic rings are almost perpendicular to each other [dihedral angle = 86.66 (7)°]. The *p*-nitro moiety is approximately co-planar with the benzene ring to which it is attached [O3–N1–C4–C5 = -175.29 (14) °] while the the one in the *o*-position is twisted [O5–N2–C2–C3 = -40.76 (17)°].

Fig. 2 shows and overlay diagram of (I) and the inverted molecule of (II) (Zukerman-Schpector *et al.* 2014). Clearly, the two molecules adopt very similar conformations.

In the crystal packing, molecules associate *via* C—H···O inter­actions, whereby the O atoms of the *o*-nitro group inter­act with H derived from a benzene or a phenyl ring, leading to a supra­molecular helical chain along [0 1 0]; Fig. 2. There also N···O5 inter­actions [2.8461 (19) Å], *i.*e. nitro···nitro inter­actions (Daszkiewicz, 2013), that link the chains into a layer. Layers stack along [0 0 1] without specific inter­molecular inter­actions between them, Fig. 3.

### S5. Synthesis and crystallization

Potassium carbonate (232 mg, 2.4 mmol) and 2,4-di­nitro­phenol (368 mg, 2 mmol) were added to a solution of 4-nitro­phenol (1.53 g, 11 mmol) and 3-bromo­methyl-4-phenyl-3-buten-2-one (478 mg, 2 mmol) in dry acetone (4 mL). The reaction mixture was stirred for 16 hours and the filtered. The solvent was evaporated and the residue was purified by column chromatography (hexane/EtOAc, 80:20) to afford the product as a colourless solid in 75% yield. The product was recrystallized by slow evaporation of a 2:1 di­chloro­methane/hexane mixture. *M*.pt: 442.2–442.8 K. ^1^H NMR (CD_2_Cl_2_, 400 MHz): δ 2.52 (3*H*, s), 5.12 (2*H*, s), 7.41 (d, 1*H*, *J* = 9.2 Hz), 7.43-7.56 (5*H*, m), 7.99 (1*H*, s), 8.42 (dd, 1*H*, *J* = 2.8, 9.2 Hz), 8.72 (d, 1*H*, *J* = 2.8 Hz). ^13^C NMR (CD_2_Cl_2_, 150 MHz): δ 26.2, 64.7, 116.1, 122.3, 129.5, 129.7, 130.2, 130.8, 134.5, 134.6, 139.8, 140.9, 148.1, 157.0, 198.4.

### S6. Refinement

Carbon-bound H-atoms were placed in calculated positions (C—H 0.93 to 0.97 Å) and were included in the refinement in the riding model approximation, with *U_iso_*(H) = 1.2*U_eq_*(C) and 1.5*U_eq_*(methyl-C).

### Crystal data

**Table:**

C_17_H_14_N_2_O_6_	*F*(000) = 712
*M**_r_* = 342.30	*D*_x_ = 1.399 Mg m^−^^3^
Monoclinic, *P*2_1_/*c*	Mo *K*α radiation, λ = 0.71073 Å
Hall symbol: -P 2ybc	Cell parameters from 6161 reflections
*a* = 12.8459 (6) Å	θ = 2.9–25.3°
*b* = 7.6983 (4) Å	µ = 0.11 mm^−^^1^
*c* = 19.4283 (8) Å	*T* = 290 K
β = 122.254 (2)°	Irregular, colourless
*V* = 1624.82 (14) Å^3^	0.66 × 0.45 × 0.28 mm
*Z* = 4	

### Data collection

**Table:**

Bruker Kappa APEXII CCD diffractometer	2957 independent reflections
Radiation source: fine-focus sealed tube	2630 reflections with *I* > 2σ(*I*)
Graphite monochromator	*R*_int_ = 0.019
ω and φ scans	θ_max_ = 25.3°, θ_min_ = 1.9°
Absorption correction: multi-scan (*SADABS*; Sheldrick, 1996)	*h* = −10→15
*T*_min_ = 0.699, *T*_max_ = 0.745	*k* = −7→9
10435 measured reflections	*l* = −23→19

### Refinement

**Table:**

Refinement on *F*^2^	Secondary atom site location: difference Fourier map
Least-squares matrix: full	Hydrogen site location: inferred from neighbouring sites
*R*[*F*^2^ > 2σ(*F*^2^)] = 0.037	H-atom parameters constrained
*wR*(*F*^2^) = 0.101	*w* = 1/[σ^2^(*F*_o_^2^) + (0.047*P*)^2^ + 0.4196*P*] where *P* = (*F*_o_^2^ + 2*F*_c_^2^)/3
*S* = 1.04	(Δ/σ)_max_ < 0.001
2957 reflections	Δρ_max_ = 0.20 e Å^−^^3^
228 parameters	Δρ_min_ = −0.21 e Å^−^^3^
0 restraints	Extinction correction: *SHELXL97* (Sheldrick, 2008), Fc^*^=kFc[1+0.001xFc^2^λ^3^/sin(2θ)]^-1/4^
Primary atom site location: structure-invariant direct methods	Extinction coefficient: 0.0323 (19)

### Special details

**Table:**

Geometry. All s.u.'s (except the s.u. in the dihedral angle between two l.s. planes) are estimated using the full covariance matrix. The cell s.u.'s are taken into account individually in the estimation of s.u.'s in distances, angles and torsion angles; correlations between s.u.'s in cell parameters are only used when they are defined by crystal symmetry. An approximate (isotropic) treatment of cell s.u.'s is used for estimating s.u.'s involving l.s. planes.
Refinement. Refinement of *F*^2^ against ALL reflections. The weighted *R*-factor *wR* and goodness of fit *S* are based on *F*^2^, conventional *R*-factors *R* are based on *F*, with *F* set to zero for negative *F*^2^. The threshold expression of *F*^2^ > 2σ(*F*^2^) is used only for calculating *R*-factors(gt) *etc*. and is not relevant to the choice of reflections for refinement. *R*-factors based on *F*^2^ are statistically about twice as large as those based on *F*, and *R*- factors based on ALL data will be even larger.

### Fractional atomic coordinates and isotropic or equivalent isotropic displacement parameters (Å^2^)

**Table:**

	*x*	*y*	*z*	*U*_iso_*/*U*_eq_	
C1	0.93401 (11)	0.15407 (17)	0.37127 (8)	0.0384 (3)	
C2	0.99377 (11)	0.00425 (16)	0.36899 (7)	0.0364 (3)	
C3	1.08837 (11)	0.01022 (18)	0.35523 (8)	0.0409 (3)	
H3	1.1239	−0.0912	0.3510	0.049*	
C4	1.12866 (12)	0.17061 (19)	0.34788 (8)	0.0436 (3)	
C5	1.07599 (14)	0.3221 (2)	0.35327 (9)	0.0509 (4)	
H5	1.1062	0.4292	0.3496	0.061*	
C6	0.97836 (14)	0.31403 (18)	0.36410 (10)	0.0494 (4)	
H6	0.9418	0.4161	0.3666	0.059*	
C7	0.76361 (13)	0.28733 (18)	0.36901 (10)	0.0463 (3)	
H7A	0.7331	0.3422	0.3167	0.056*	
H7B	0.8140	0.3705	0.4115	0.056*	
C8	0.65850 (12)	0.22873 (19)	0.37591 (9)	0.0437 (3)	
C9	0.68382 (13)	0.2244 (2)	0.46004 (9)	0.0503 (4)	
C10	0.58981 (16)	0.1491 (3)	0.47503 (11)	0.0656 (5)	
H10A	0.6229	0.1458	0.5325	0.098*	
H10B	0.5694	0.0334	0.4534	0.098*	
H10C	0.5171	0.2199	0.4488	0.098*	
C11	0.54588 (13)	0.18897 (19)	0.31208 (9)	0.0464 (3)	
H11	0.4877	0.1628	0.3251	0.056*	
C12	0.50092 (13)	0.18058 (18)	0.22527 (9)	0.0447 (3)	
C13	0.57329 (14)	0.1286 (2)	0.19522 (9)	0.0502 (4)	
H13	0.6557	0.1012	0.2312	0.060*	
C14	0.52360 (15)	0.1174 (2)	0.11257 (10)	0.0568 (4)	
H14	0.5729	0.0830	0.0933	0.068*	
C15	0.40169 (16)	0.1568 (2)	0.05828 (10)	0.0596 (4)	
H15	0.3690	0.1496	0.0026	0.071*	
C16	0.32823 (15)	0.2069 (2)	0.08653 (10)	0.0613 (4)	
H16	0.2458	0.2337	0.0500	0.074*	
C17	0.37725 (14)	0.2172 (2)	0.16927 (10)	0.0539 (4)	
H17	0.3268	0.2492	0.1880	0.065*	
N1	1.22919 (11)	0.1784 (2)	0.33327 (8)	0.0572 (4)	
N2	0.95589 (10)	−0.16711 (15)	0.38003 (7)	0.0443 (3)	
O1	0.83551 (8)	0.13359 (12)	0.37729 (6)	0.0459 (3)	
O2	0.78088 (11)	0.27977 (19)	0.51669 (7)	0.0716 (4)	
O3	1.26869 (11)	0.0420 (2)	0.32347 (8)	0.0767 (4)	
O4	1.26880 (12)	0.32170 (19)	0.33128 (9)	0.0825 (4)	
O5	0.95090 (13)	−0.28306 (15)	0.33557 (8)	0.0716 (4)	
O6	0.93475 (11)	−0.18635 (15)	0.43354 (8)	0.0673 (4)	

### Atomic displacement parameters (Å^2^)

**Table:**

	*U*^11^	*U*^22^	*U*^33^	*U*^12^	*U*^13^	*U*^23^
C1	0.0332 (6)	0.0384 (7)	0.0442 (7)	−0.0002 (5)	0.0209 (6)	0.0030 (5)
C2	0.0316 (6)	0.0352 (7)	0.0391 (6)	−0.0014 (5)	0.0167 (5)	0.0027 (5)
C3	0.0341 (6)	0.0469 (8)	0.0400 (7)	0.0039 (6)	0.0187 (6)	0.0018 (6)
C4	0.0338 (6)	0.0564 (9)	0.0412 (7)	−0.0039 (6)	0.0204 (6)	0.0042 (6)
C5	0.0478 (8)	0.0461 (8)	0.0605 (9)	−0.0090 (6)	0.0301 (7)	0.0067 (7)
C6	0.0496 (8)	0.0357 (7)	0.0681 (10)	0.0009 (6)	0.0349 (8)	0.0041 (7)
C7	0.0426 (7)	0.0407 (8)	0.0599 (9)	0.0080 (6)	0.0302 (7)	0.0032 (6)
C8	0.0405 (7)	0.0443 (8)	0.0518 (8)	0.0104 (6)	0.0284 (6)	0.0041 (6)
C9	0.0443 (8)	0.0550 (9)	0.0509 (8)	0.0158 (7)	0.0248 (7)	0.0081 (7)
C10	0.0647 (10)	0.0875 (13)	0.0582 (10)	0.0071 (9)	0.0419 (9)	0.0055 (9)
C11	0.0418 (7)	0.0524 (8)	0.0542 (8)	0.0061 (6)	0.0318 (7)	0.0013 (6)
C12	0.0436 (7)	0.0443 (8)	0.0517 (8)	0.0024 (6)	0.0291 (7)	0.0003 (6)
C13	0.0470 (8)	0.0546 (9)	0.0541 (8)	0.0103 (7)	0.0304 (7)	0.0040 (7)
C14	0.0668 (10)	0.0592 (10)	0.0597 (9)	0.0106 (8)	0.0440 (8)	0.0052 (8)
C15	0.0666 (10)	0.0640 (11)	0.0492 (9)	0.0053 (8)	0.0317 (8)	0.0051 (7)
C16	0.0463 (8)	0.0755 (12)	0.0553 (9)	0.0049 (8)	0.0226 (7)	0.0018 (8)
C17	0.0418 (7)	0.0650 (10)	0.0591 (9)	0.0018 (7)	0.0298 (7)	−0.0040 (7)
N1	0.0398 (7)	0.0847 (11)	0.0497 (7)	−0.0076 (7)	0.0256 (6)	0.0055 (7)
N2	0.0342 (6)	0.0364 (6)	0.0536 (7)	0.0010 (5)	0.0177 (5)	0.0058 (5)
O1	0.0380 (5)	0.0394 (5)	0.0685 (6)	0.0048 (4)	0.0339 (5)	0.0072 (4)
O2	0.0542 (7)	0.0928 (10)	0.0513 (7)	0.0044 (6)	0.0170 (6)	0.0071 (6)
O3	0.0611 (7)	0.0984 (10)	0.0919 (9)	0.0169 (7)	0.0550 (7)	0.0151 (8)
O4	0.0699 (8)	0.0988 (11)	0.0971 (10)	−0.0359 (7)	0.0567 (8)	−0.0084 (8)
O5	0.0896 (9)	0.0395 (6)	0.0671 (8)	−0.0070 (6)	0.0294 (7)	−0.0080 (5)
O6	0.0747 (8)	0.0542 (7)	0.0984 (9)	0.0033 (6)	0.0632 (8)	0.0199 (6)

### Geometric parameters (Å, º)

**Table:**

C1—O1	1.3405 (15)	C10—H10A	0.9600
C1—C6	1.3947 (18)	C10—H10B	0.9600
C1—C2	1.3993 (18)	C10—H10C	0.9600
C2—C3	1.3773 (17)	C11—C12	1.464 (2)
C2—N2	1.4610 (16)	C11—H11	0.9300
C3—C4	1.375 (2)	C12—C17	1.394 (2)
C3—H3	0.9300	C12—C13	1.3945 (19)
C4—C5	1.380 (2)	C13—C14	1.378 (2)
C4—N1	1.4638 (17)	C13—H13	0.9300
C5—C6	1.379 (2)	C14—C15	1.376 (2)
C5—H5	0.9300	C14—H14	0.9300
C6—H6	0.9300	C15—C16	1.376 (2)
C7—O1	1.4565 (16)	C15—H15	0.9300
C7—C8	1.4959 (19)	C16—C17	1.381 (2)
C7—H7A	0.9700	C16—H16	0.9300
C7—H7B	0.9700	C17—H17	0.9300
C8—C11	1.345 (2)	N1—O4	1.2242 (18)
C8—C9	1.487 (2)	N1—O3	1.2242 (19)
C9—O2	1.2176 (19)	N2—O6	1.2156 (16)
C9—C10	1.502 (2)	N2—O5	1.2198 (16)
			
O1—C1—C6	124.58 (12)	H10A—C10—H10B	109.5
O1—C1—C2	117.72 (11)	C9—C10—H10C	109.5
C6—C1—C2	117.66 (12)	H10A—C10—H10C	109.5
C3—C2—C1	122.31 (12)	H10B—C10—H10C	109.5
C3—C2—N2	117.14 (11)	C8—C11—C12	129.87 (13)
C1—C2—N2	120.55 (11)	C8—C11—H11	115.1
C4—C3—C2	118.04 (12)	C12—C11—H11	115.1
C4—C3—H3	121.0	C17—C12—C13	117.91 (13)
C2—C3—H3	121.0	C17—C12—C11	118.36 (12)
C3—C4—C5	121.58 (12)	C13—C12—C11	123.63 (13)
C3—C4—N1	118.47 (13)	C14—C13—C12	120.53 (14)
C5—C4—N1	119.95 (13)	C14—C13—H13	119.7
C6—C5—C4	119.75 (13)	C12—C13—H13	119.7
C6—C5—H5	120.1	C15—C14—C13	120.61 (14)
C4—C5—H5	120.1	C15—C14—H14	119.7
C5—C6—C1	120.54 (13)	C13—C14—H14	119.7
C5—C6—H6	119.7	C16—C15—C14	119.88 (15)
C1—C6—H6	119.7	C16—C15—H15	120.1
O1—C7—C8	107.14 (11)	C14—C15—H15	120.1
O1—C7—H7A	110.3	C15—C16—C17	119.79 (15)
C8—C7—H7A	110.3	C15—C16—H16	120.1
O1—C7—H7B	110.3	C17—C16—H16	120.1
C8—C7—H7B	110.3	C16—C17—C12	121.26 (14)
H7A—C7—H7B	108.5	C16—C17—H17	119.4
C11—C8—C9	120.33 (12)	C12—C17—H17	119.4
C11—C8—C7	124.35 (13)	O4—N1—O3	123.64 (13)
C9—C8—C7	115.26 (13)	O4—N1—C4	117.88 (15)
O2—C9—C8	120.24 (14)	O3—N1—C4	118.49 (13)
O2—C9—C10	120.09 (14)	O6—N2—O5	124.41 (13)
C8—C9—C10	119.67 (14)	O6—N2—C2	118.65 (12)
C9—C10—H10A	109.5	O5—N2—C2	116.90 (12)
C9—C10—H10B	109.5	C1—O1—C7	117.86 (10)
			
O1—C1—C2—C3	173.75 (11)	C8—C11—C12—C17	−150.23 (16)
C6—C1—C2—C3	−3.9 (2)	C8—C11—C12—C13	33.5 (2)
O1—C1—C2—N2	−5.24 (18)	C17—C12—C13—C14	1.2 (2)
C6—C1—C2—N2	177.08 (12)	C11—C12—C13—C14	177.53 (15)
C1—C2—C3—C4	3.50 (19)	C12—C13—C14—C15	−0.2 (3)
N2—C2—C3—C4	−177.48 (11)	C13—C14—C15—C16	−0.4 (3)
C2—C3—C4—C5	−0.6 (2)	C14—C15—C16—C17	−0.1 (3)
C2—C3—C4—N1	179.98 (11)	C15—C16—C17—C12	1.1 (3)
C3—C4—C5—C6	−1.8 (2)	C13—C12—C17—C16	−1.7 (2)
N1—C4—C5—C6	177.66 (13)	C11—C12—C17—C16	−178.17 (15)
C4—C5—C6—C1	1.3 (2)	C3—C4—N1—O4	−176.08 (13)
O1—C1—C6—C5	−176.05 (13)	C5—C4—N1—O4	4.5 (2)
C2—C1—C6—C5	1.5 (2)	C3—C4—N1—O3	4.2 (2)
O1—C7—C8—C11	−94.16 (16)	C5—C4—N1—O3	−175.29 (14)
O1—C7—C8—C9	88.75 (14)	C3—C2—N2—O6	137.34 (13)
C11—C8—C9—O2	−171.36 (15)	C1—C2—N2—O6	−43.62 (17)
C7—C8—C9—O2	5.9 (2)	C3—C2—N2—O5	−40.76 (17)
C11—C8—C9—C10	9.5 (2)	C1—C2—N2—O5	138.28 (13)
C7—C8—C9—C10	−173.26 (13)	C6—C1—O1—C7	5.4 (2)
C9—C8—C11—C12	−178.44 (14)	C2—C1—O1—C7	−172.16 (12)
C7—C8—C11—C12	4.6 (2)	C8—C7—O1—C1	178.57 (11)

### Hydrogen-bond geometry (Å, º)

**Table:**

*D*—H···*A*	*D*—H	H···*A*	*D*···*A*	*D*—H···*A*
C6—H6···O5^i^	0.93	2.41	3.1375 (18)	135
C16—H16···O6^ii^	0.93	2.58	3.292 (3)	134

Symmetry codes: (i) *x*, *y*+1, *z*; (ii) −*x*+1, *y*+1/2, −*z*+1/2.
