# Supplementary figures and images for: Crystal structure of (3E)-3-(2,4-di­nitro­phen­oxy­meth­yl)-4-phenyl­but-3-en-2-one
Source: Acta Crystallogr Sect E Struct Rep Online. 2014 Aug 23;70(Pt 9):o1051–2. doi: 10.1107/S1600536814018819 (PMC4186202; doi:10.1107/S1600536814018819)

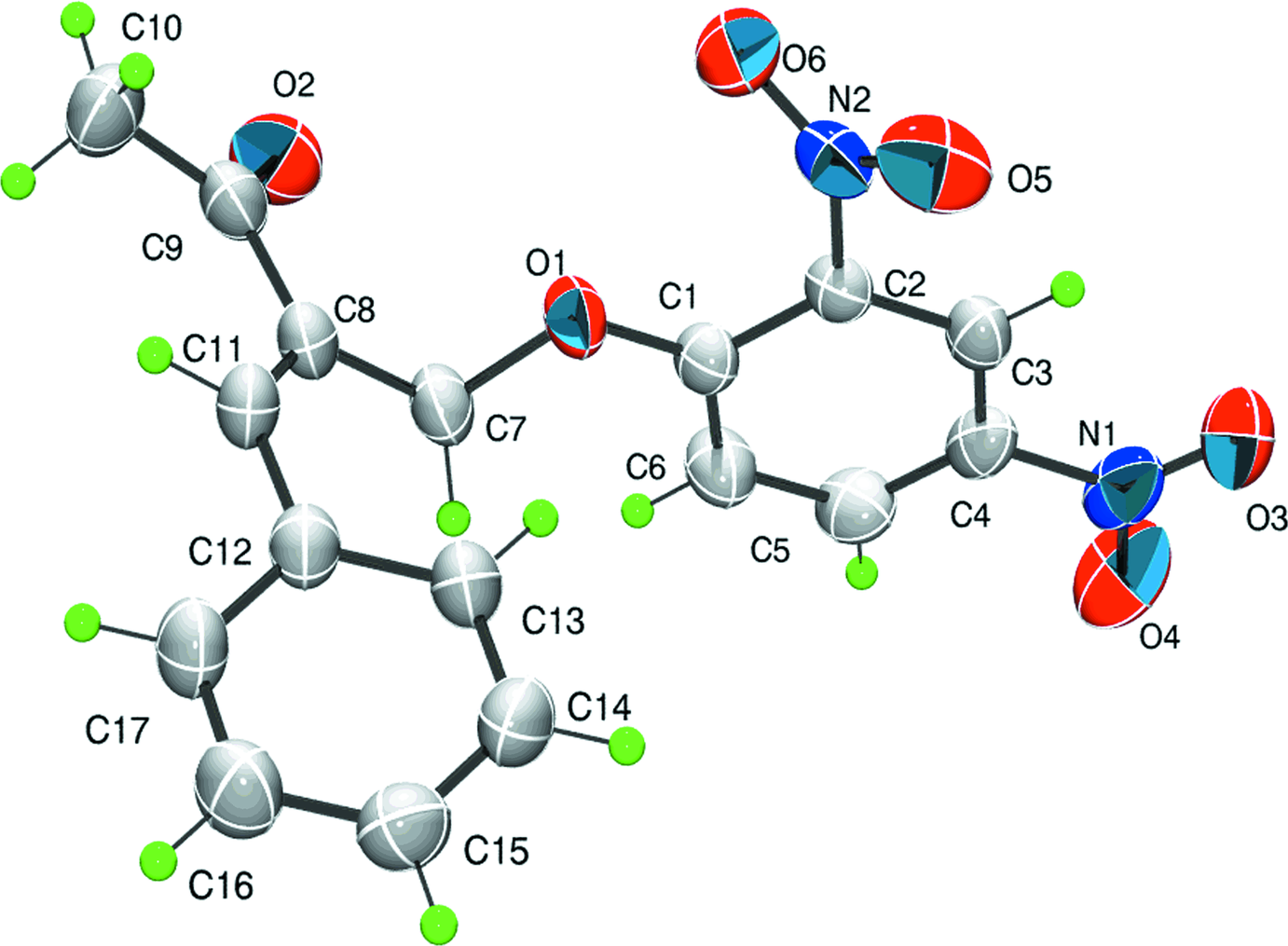

Supplement: Supplementary file 4 [file e-70-o1051-fig1.tif]

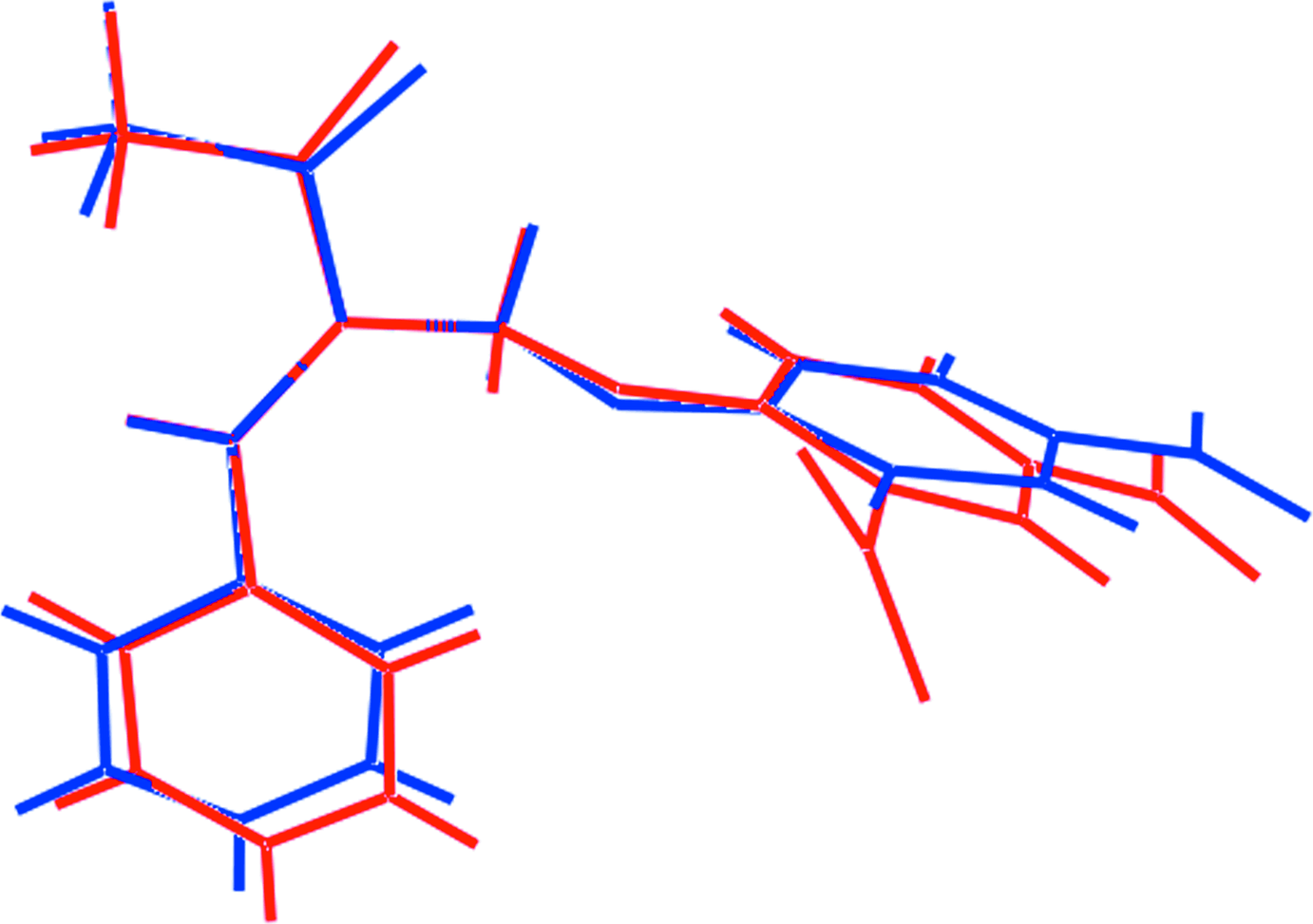

Supplement: Supplementary file 5 [file e-70-o1051-fig2.tif]

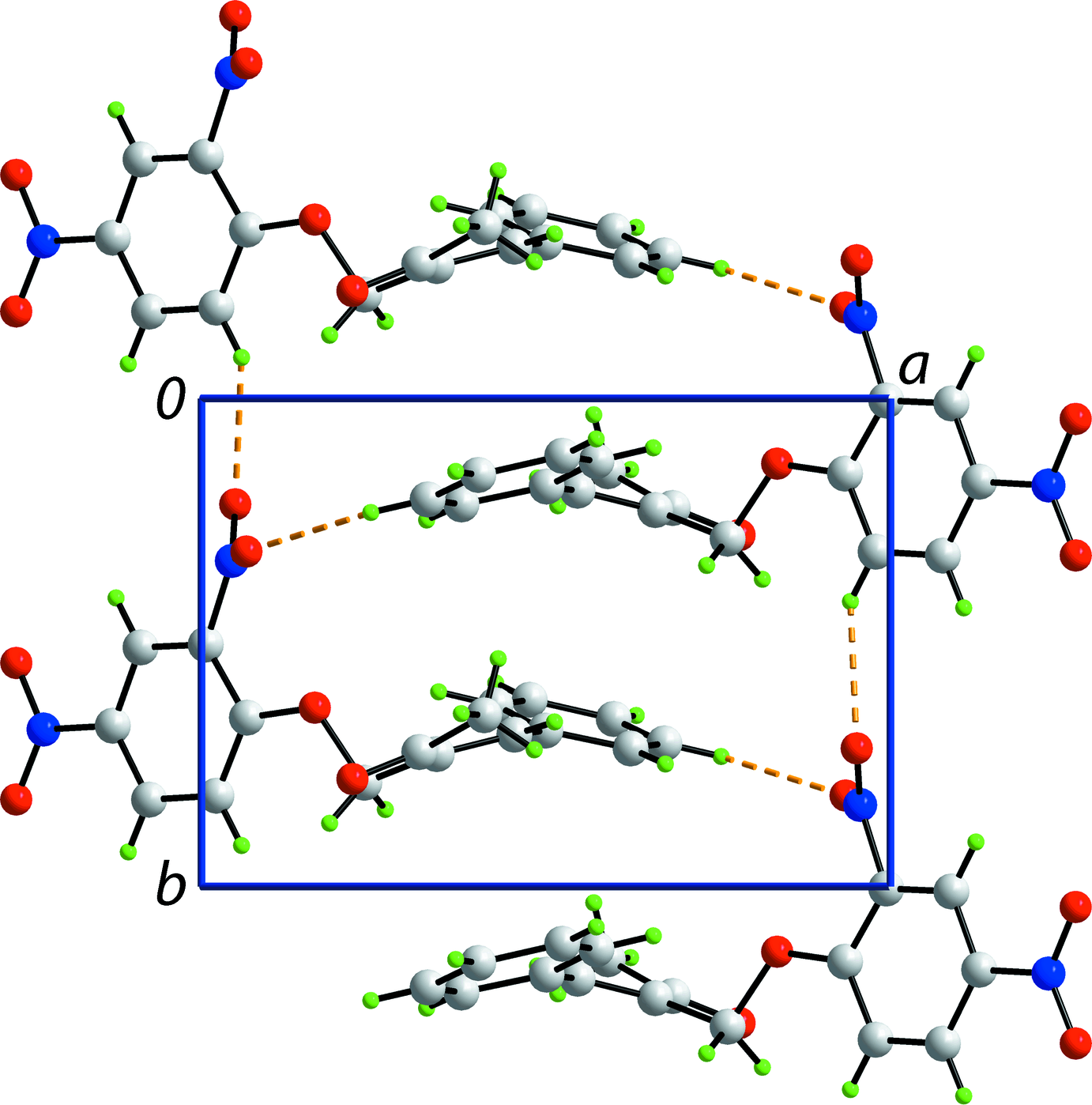

Supplement: Supplementary file 6 [file e-70-o1051-fig3.tif]

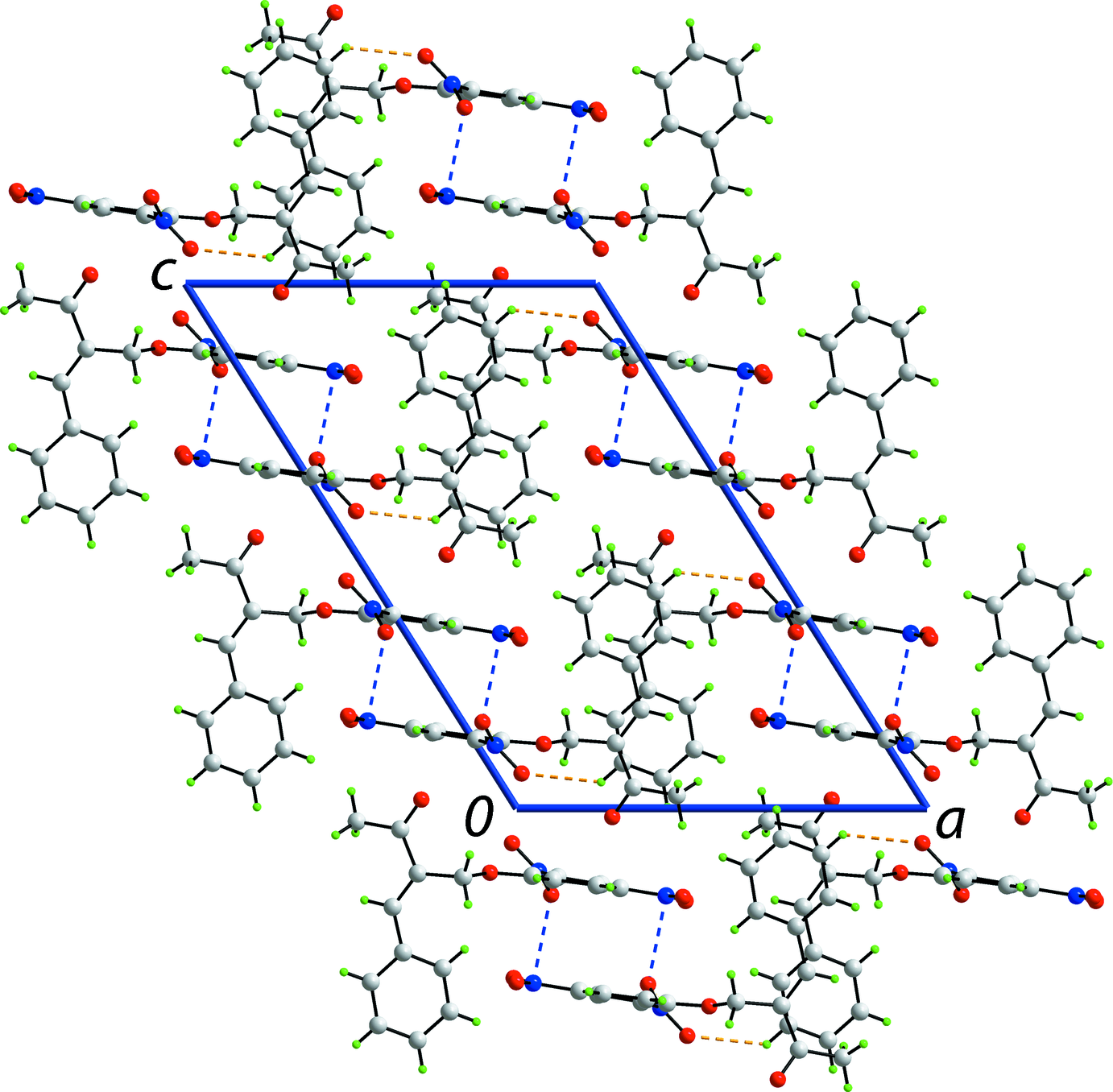

Supplement: Supplementary file 7 [file e-70-o1051-fig4.tif]
